# A Smartphone-based Diffusometric Immunoassay for Detecting C-Reactive Protein

**DOI:** 10.1038/s41598-019-52285-4

**Published:** 2019-11-20

**Authors:** Chih-Shen Chuang, Chih-Zong Deng, Yi-Fan Fang, Hong-Ren Jiang, Pao-Wei Tseng, Horn-Jiunn Sheen, Yu-Jui Fan

**Affiliations:** 1 Institute of Applied Mechanics, National Taiwan University, 1 Roosevelt Road, Sec. 4, Taipei, 10617 Taiwan; 2 School of Biomedical Engineering, Taipei Medical University, 250 Wuxing St., Taipei, 11031 Taiwan; 3 Department of Mechanical Engineering, The University of Tokyo, 7 Chome-3-1 Hongo, Bunkyō, Tokyo, 113-8654 Japan; 4 International PhD Program for Biomedical Engineering, Taipei Medical University, 250 Wuxing St., Taipei, 11031 Taiwan; 5 Graduate Institute of Biomedical Optomechatronics, Taipei Medical University, 250 Wuxing St., Taipei, 11031 Taiwan

**Keywords:** Biochemical assays, Health care

## Abstract

In this study, we developed a portable smartphone-based diffusometry for analyzing the C-reactive protein (CRP) concentration. An optimized fluorescence microscopic add-on system for a smartphone was used to image the 300 nm fluorescent beads. Sequential nanobead images were recorded for a period and the image data were used for fluorescence correlation spectrometric (FCS) analysis. Through the analysis, the nanobeads’ diffusion coefficient was obtained. Further, the diffusion coefficients of the anti-CRP-coated nanobeads, which were suspended in the samples with various CRP concentrations, were estimated using smartphone-based diffusometry. After 10 min of reaction, the anti-CRP-coated nanobeads in a higher CRP concentration solution led to a lower diffusion coefficient. Based on the experiments, a linear sensing range of 1~8 µg/mL was found.

## Introduction

C-Reactive protein (CRP), an acute-phase protein, is an important risk factor for atherosclerosis and coronary heart disease^1^. Moreover, CRP is a sensitive biomarker of inflammation and has proven useful as a prognostic indicator of inflammation, infections, tissue necrosis, surgery, burns, cancer, cardiovascular diseases, and coronary heart disease risk^2–5^. The CRP concentration can increase by up to three orders of magnitude when a tissue becomes inflamed^6,7^. Therefore, an instantaneous check of the CRP level is critical for clinical diagnoses.

Several common technologies are often used for CRP measurement nowadays, such as enzyme-linked immunosorbent assay (ELISA), rapid immunodiffusion, nephelometry, immunoturbidimetry, and visual agglutination with detection ranges of 0.1~0.2 µg/mL. These techniques have different disadvantages including large sample consumption, professional training required, and being time consuming, although they are reliable for clinical diagnoses. In particular, the time-consumption issue means that current approaches might not be the best choice for detecting or tracking the progress of a rise in the CRP level which increases at around 6 hours after a stimulus^8^.

Several approaches that are able to achieve label-free CRP sensing for rapid diagnoses, e.g. surface plasmon resonance, SPR^9^, quartz crystal microbalance, QCM^10^, piezoelectric micro-cantilever^11^, electrochemistry^8^, electrochemical impedance spectroscopy^12^, micro-particle-tracking-velocimetry, Micro-PTV^13,14^, and fiber Bragg gratings (FBGs) have been described in the literature^15^. However, most of these technologies require a bulk facility and may have barriers to becoming portable devices for point-of-care (POC) tests.

Smartphone that have a high-quality camera and an operating system have been becoming a popular sensing device in recent years, especially in POC analyses. In the health sector, the smartphones can be used as detectors due to the high-quality camera. Furthermore, the smartphone can be an interface with add-on analytical instruments because of Wi-Fi, Bluetooth, and microUSB technologies^16^. Currently, smartphone sensing techniques are mainly classified into three parts: colorimetry, spectroscopic sensing, and an electrochemical sensor. Several studies pointed out that colorimetric sensor systems can be used to detect blood hematocrit^17^, alkaline phosphatase activity in milk^18^, and human CRP levels^19^. In the spectroscopic sensing sector, Zangheri *et al*. pointed out that salivary cortisol can be detected more easily and faster using a smartphone and a chemiluminescent lateral-flow immunoassay (CL-LFIA)^20^. Xiao *et al*. developed a compact smartphone-based device to read gold nanoparticle-enhanced test strips^21^. A SPR platform with biological assays can also be based on a smartphone system. Guner *et al*. demonstrated that an immobilized layer of rabbit anti-mouse (RAM) immunoglobulin G (IgG) can capture the mouse IgG antibody^22^. 17-β-Estradiol in water can be detected using smartphone imaging-based fluorescence microscopy^23^. A smartphone-based device with an electrochemical chip can also be used to verify one’s gender^24^. Detection of bovine serum albumin (BSA), thrombin, and 2,4,6-trinitrotoluene (TNT) can also use electrochemical impedance spectroscopy (EIS) with a smartphone^25^. Chen *et al*. also reported a paper-based bipolar electrode system that can detect glucose in phosphate-buffered saline (PBS) solutions and artificial urine samples^26^. Zhu *et al*. developed cellphone-based flow cytometry by integrating a microfluidic channel and smartphone to count and analyze human blood^27^.

An ELISA and other photometric methods show the potential to become portable by using a smartphone as a reader. Recently, several efforts were devoted to achieve a paper/microfluidic-based colorimetric assay coupled with a smartphone readout. Hsu *et al*. delivered an intraocular vascular endothelial growth factor (VEGF) diagnosis using a paper-based ELISA and smartphone readout^28^. Wu *et al*. used paper-based Dot-ELISA integrated with a reagent storage microfluidic system and smartphone imaging system to detect influenza A^29^. Wang *et al*. detected uric acid and glucose through a paper-based colorimetric assay and used a smartphone as a readout^30^. Wang *et al*. used a microfluidic-based ELISA and imaging with a cellphone to detect the HE4 biomarker in urine for screening ovarian cancer^31^. Huang *et al*. used a paper-based colorimetric assay to detect single-stranded (ss)DNA for cancer diagnoses, and used gold nanoparticles to enhance the signals from the cellphone readouts^32^. A paper-based ELISA can reduce sample consumption; however, the time consumption issue due to multiple-step processes has still not been resolved. Further, an ELISA also has image distortion and chromatic aberration issues that need to be calibrated especially when using a cellphone as a reader.

In our previous study, the CRP quantification by using micro-PTV was developed. The anti-CRP modified nanobeads’ movements can be individually measured. Thereafter, the Brownian velocities and the diffusion coefficients can be statistically analyzed. When the anti-CRP modified nanobeads suspending in different CRP concentration solution, the nanobeads’ decreasing rate will be varied, and the equilibrium Brownian velocity can be found with 10 minute reaction. However, the data analysis computation is too much to be implemented by a smartphone.

**Figure 1:**
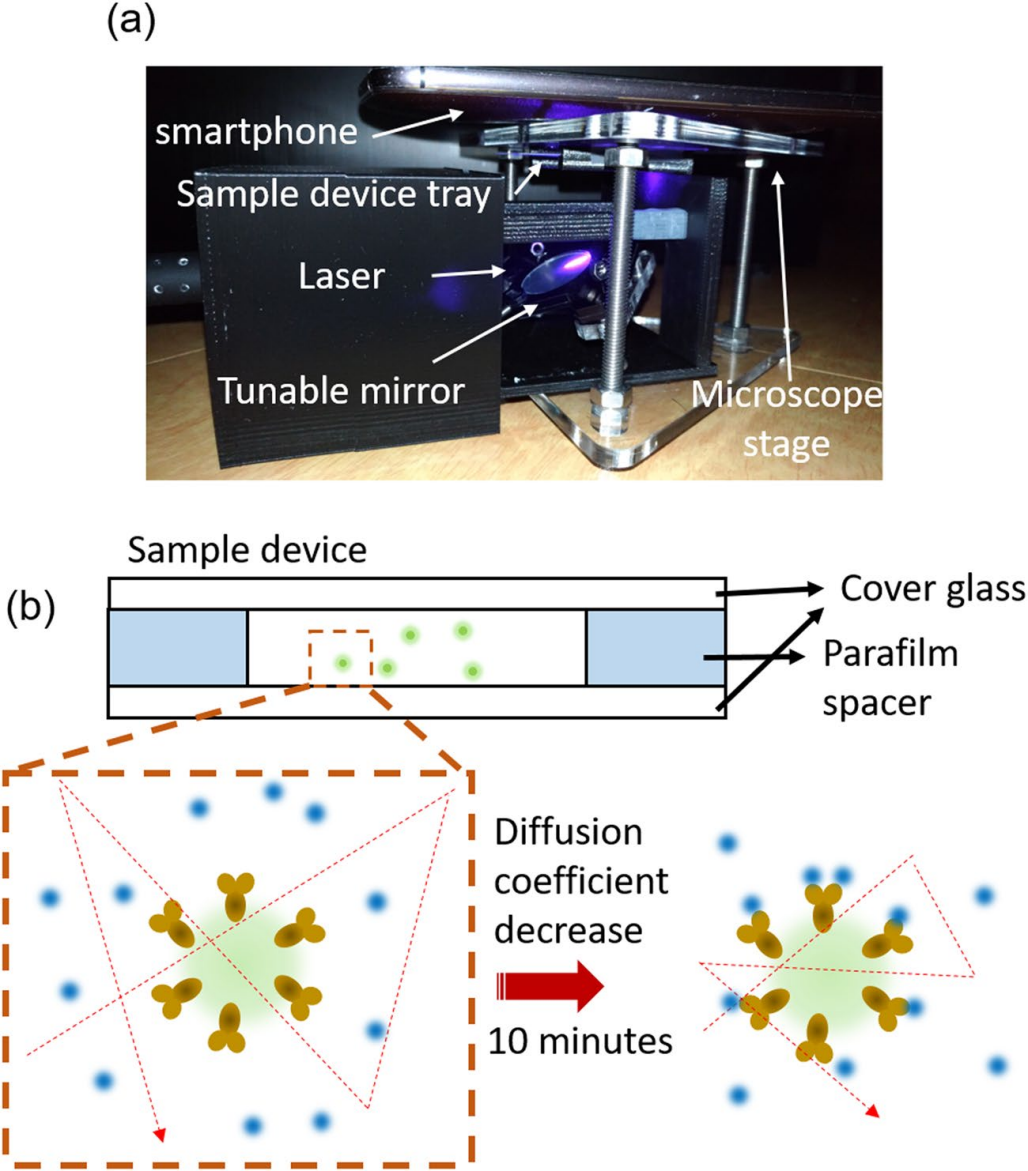
(**a**) Smartphone add-on system for analyzing diffusion coefficient of nanobeads. (**b**) The anti-CRP modified nanobeads interacted with CRP in 10 min in a micro-chamber, and the nanobeads’ diffusion coefficient can be carried out in real-time.

Herein, we developed a novel smartphone-based diffusometry which can be used to measure the Brownian diffusion coefficient of the nanobeads for label-free sensing of CRP concentrations as Fig. 1. An achromatic fluorescent microscopic add-on system for a smartphone was developed to obtain the high-resolution fluorescence images of the nanobeads. The sequence of fluorescent nanobead images can be used to analyze the nanobeads’ diffusion coefficients. To compute the nanobeads’ diffusion coefficient by using a smartphone, a new method was developed which was based on the algorithm of fluorescence correlation spectroscopy (FCS). Nanobead diffusion coefficients correspond to their size and vary with different amounts of CRP conjugation. When the antibody-coated nanobeads are suspended in a CRP solution and interact with CRP, the nanobeads’ Brownian diffusivity will decrease. Samples with a higher CRP concentration will exhibit lower diffusion coefficients of suspended anti-CRP-coated nanobeads in the sensing range.

## Results

### Optical system

The smartphone-based diffusometry is consisted of a commercial available smartphone and an add-on system of a high-resolution fluorescent microscope, and a diffusometric algorithm is used for data reduction.

**Figure 2:**
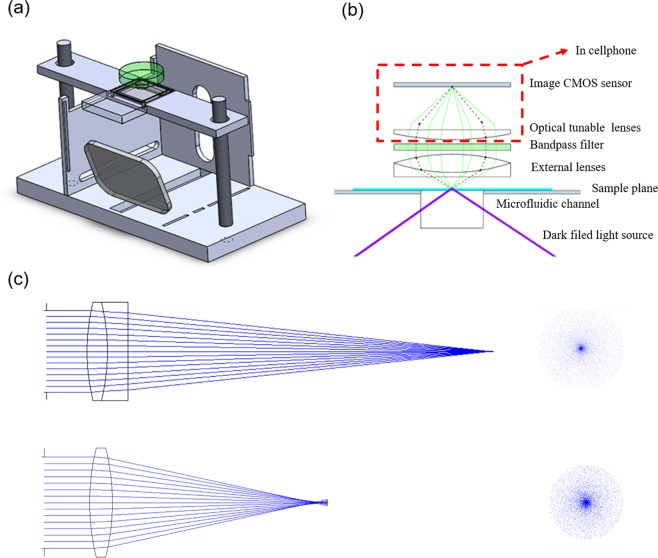
(**a**) Schematic optical system of the high-resolution fluorescent microscope. (**b**) Setup of the optical system. (**c**) The ray tracing results compared to the achromatic lens set with simple dual convex lens. Schematics of the sensing mechanism.

Figure 2(a,b) show the optical system and the high-resolution smartphone-based fluorescent microscope for imaging a population of 335 nm beads. The suitcase of the add-on system and the sample device tray were designed by 3D computer aid design (CAD) software and built by using 3D printer to print the 3D structure of polylactic acid (PLA). The stage of the smartphone-based microscope was made by laser-cutting acrylic and supported by three screws.

The smartphone was placed on a stage, and its camera was aligned with a set of lenses embedded in the stage. A emission chromatic filter (ET525/50 m, Chroma technology), which is hard coated by sputter and the wavelength is 525 nm with bandwidth of 50 nm, was used and placed between the phone camera and the magnification lenses to filter out the background noise. In front of the lens, a sample device tray was designed to hold the samples, which were sandwiched between two 22 × 22-mm, 130 μm thick, cover glasses with parafilm spacer. A collimated 5 mW laser diode with wavelength of 405 nm was used to illuminate the samples through a tunable mirror to adjust the incident angle. The cage of the tunable mirror was also made by two layer of the laser-cutting acrylic. The two acrylic plates were fixed by two springs as the recover function. The two screws were also used to fine tune the lift angels in x and y direction.

To reduce the spherical and chromatic aberrations, an achromatic lens set consisting of a dual-convex lens and a concave-plane lens, was used to image the fluorescent nanobeads. The ray tracing results in Fig. 2(a) show that the achromatic lens set had less aberration compared to the simple dual-convex lens when collimated light passed through. In this system, the fluorescent nanobeads were imaged through the achromatic lens set, and the magnification was 5X, as confirmed by a calibration ruler. A sample video result of Fig. 2(b), which was taken by a smartphone (Zenfone Zoom, ASUS). This smartphone carrys a 3X optical zoom lens camera to better record the real-time Brownian motion of the nanobeads. The highest magnification of 15X can be obtained to provide high spatial resolution for analyzing nanobeads’ movement. The antibody modified immunobeads will interact with the antigens when suspending in a sample solution, and results in that the immunobeads’ Brownian diffusion decrease as depicted in Fig. 1(b).

### Sample preparations

**Figure 3:**
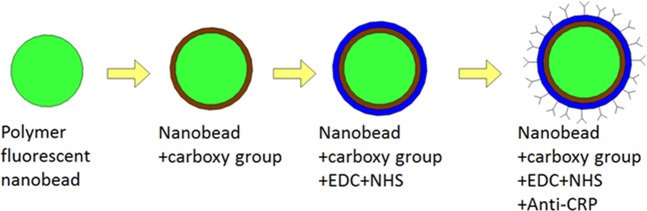
Preparation procedures for nanobeads with anti-CRPs.

Fluorescent polystyrene beads (MerckTM XC030) with a diameter of 335 nm, modified with carboxyl functional group (COOH^−^) on the surface, was used in this experiment. The optimal excitation and emission wavelengths are 475 and 525 nm, respectively. The anti-CRP was conjugated with the nanobeads following a well-known protocol for efficient two-step coupling using 1-ethyl-3-(3-dimethylaminopropyl) carbodiimide (EDC) and N-hydroxysuccinimide (NHS) as Fig. 3. The density of the beads was approximately 1.05 g/cm^3^, so that settlement force of the nanobeads could be neglected in a short period. EDC reacted with the nanobeads’ carboxyl groups in 2-(N-morpholino)ethanesulfonic acid (MES) buffer at pH 5.5. NHS was used to stabilize EDC, because EDC is a water-soluble carbodiimide. Subsequently, the anti-CRP, goat anti-human CRP IgG (Sigma C8284), was conjugated onto the nanobeads by linking the carboxyl group of EDC-NHS to the amino groups of anti-CRP. A shaker and porous membrane films with different aperture sizes were used to reduce the self-assembling of particles and filter out particle clusters. The individual antibody-coated nanobeads without clustering can be collected using those apparatuses mentioned above. The prepared anti-CRP-coated nanobeads were suspended in phosphate-buffered saline (PBS) for storage. Reactors for antibody-coated nanobeads and antigen interaction were fabricated by sandwiching parafilm spacer between two cover glasses. The reactor was design as a circular micro-chambers with diameter of 12 mm.

### Fluorescence correlation spectroscopy

The diffusometry is an approach to measure the diffusion coefficient of the molecules or the nanoparticles. Typical diffusometric approaches use dynamic light scattering (DLS) and fluorescence correlation spectroscopy (FCS). FCS is, in a way, the fluorescent counterpart to DLS, which is implemented with coherent light, instead of fluorescence (incoherent emissions). In this study, smartphone-based FCS was developed to estimate the diffusivity of 300 nm nanobeads by analyzing a sequence of nanobead images. FCS uses a correlation analysis of fluctuations in the fluorescence intensity. The fluctuations in the intensity are correlated with the diffusivity of the molecules in solution. In this study, the fluorescence emitted from an interrogation space in a solution containing a small number of fluorescent nanobeads was recorded by a smartphone. Because of the nanobeads’ Brownian motion, the fluorescence intensity fluctuated. This means that the number of nanobeads in the interrogation space randomly changes around an average number. The analysis gives an average number of fluorescent nanobeads and an average diffusion time, as the particles pass through the interrogation space.

To implement the FCS analysis, the fluorescence intensity function of the interrogation, *I*(t), was first recorded. The autocorrelation function, *G*(t), can be written as

1 $$G({\rm{\tau }})=\frac{\langle \delta I({\rm{t}})\delta I({\rm{t}}+{\rm{\tau }})\rangle }{{\langle I({\rm{t}})\rangle }^{2}}=\frac{\langle I({\rm{t}})I({\rm{t}}+{\rm{\tau }})\rangle }{{\langle I({\rm{t}})\rangle }^{2}}-1;$$

which is a correlation of a time series with itself shifted by time τ, where $\delta I({\rm{t}})=I({\rm{t}})-\langle I(t)\rangle$ is the deviation from the mean intensity. The correlation at τ = 0, *G*(0), is related to the average number of the nanobeads in the interrogation space.

The FCS experiment is for 3D diffusion of the nanobeads or the molecules, for which the autocorrelation is

2 $$G({\rm{\tau }})={\rm{G}}({\rm{0}})\frac{1}{(1+({\rm{\tau }}/{{\rm{\tau }}}_{{\rm{D}}})){(1+{a}^{-2}({\rm{\tau }}/{{\rm{\tau }}}_{{\rm{D}}}))}^{1/2}}+{\rm{G}}(\infty );$$

where *a* is the ratio of the depth of field to the radial e^−2^ radii of the interrogation area. τ_D_ is the characteristic residence time, which corresponds to the diffusion time, and is

3 $${\tau }_{D}=\frac{{a}^{2}}{4D},$$

where *D* is the diffusion coefficient, and can be written as

4 $$D=\frac{kT}{6\pi \mu r};$$

where k is the Boltzmann constant, *k* = 1.3805 × 10^−23^ J/K, *T* is the absolute temperature of the fluid, *µ* is the solvent viscosity, and *r* is the particle radius. In this experiment, the diffusion time was used to estimate the diffusion coefficient, which is thus sensitive to the variations in nanobead sizes due to the amount of CRP bound onto the nanobeads.

**Figure 4:**
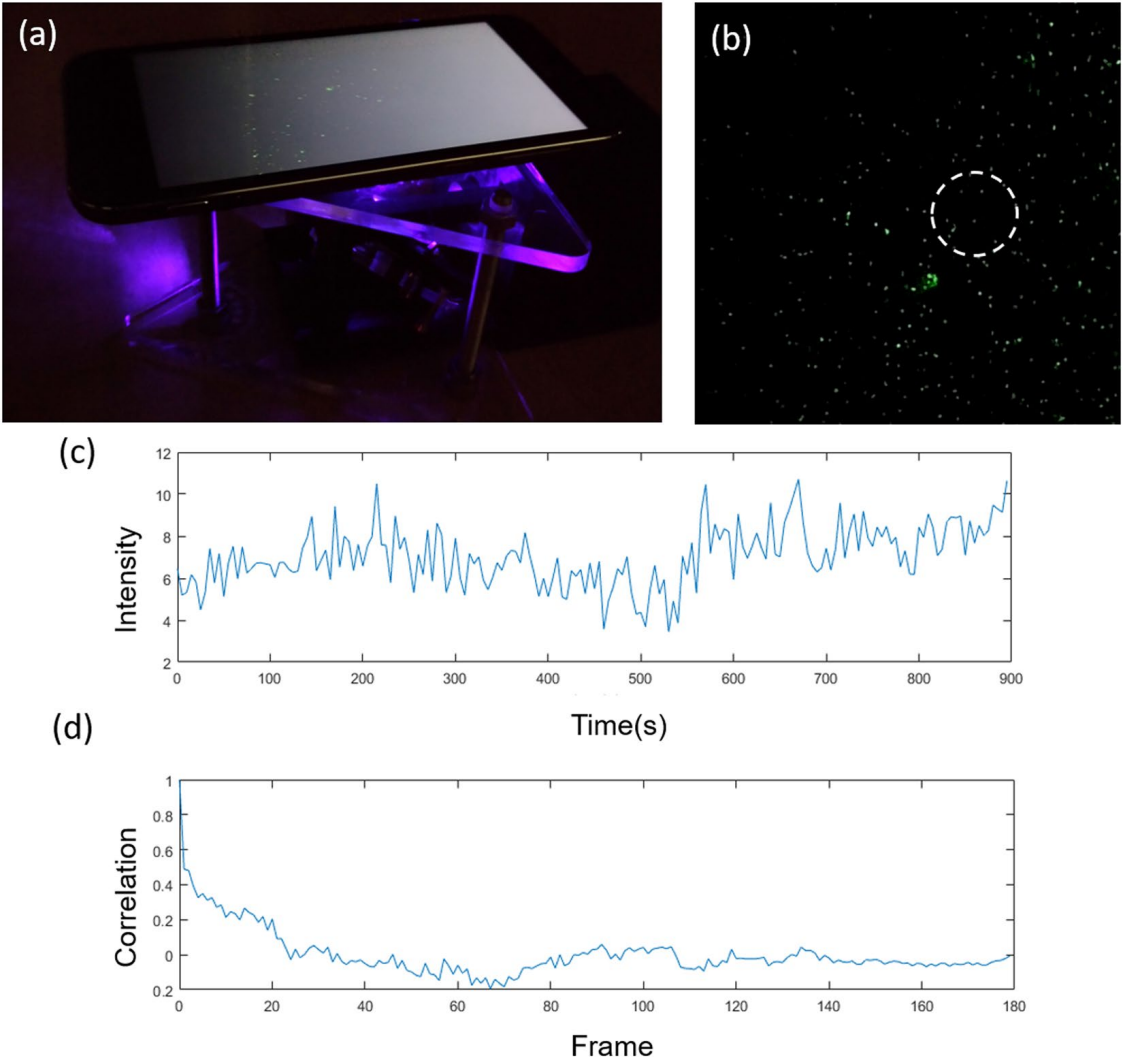
(**a**) Smartphone-based microscope for imaging 300 nm fluorescent beads (**b**) A video of the Brownian motion of the nanobeads. The interrogation window is shown as a dashed-line circle. (**c**) The intensity variation, *I*(t), in the interrogation window. (**d**) The autocorrelation function, *G*(τ), in the interrogation window.

Based on the video and FCS algorithm, the diffusion coefficient of the 335 nm fluorescent beads was able to be estimated. First, we selected an interrogation area in the video as the white dashed-line circle shown in Fig. 4(a,b), and we recorded the intensity variation in the area as *I*(t) as shown in Fig. 4(c). *G*(τ) can be found based on Eq. (1) and is shown in Fig. 4(d). Further, the diffusion time and the diffusion coefficient can be found by using Eqs (2–4).

### Detection of different CRP concentration

**Figure 5:**
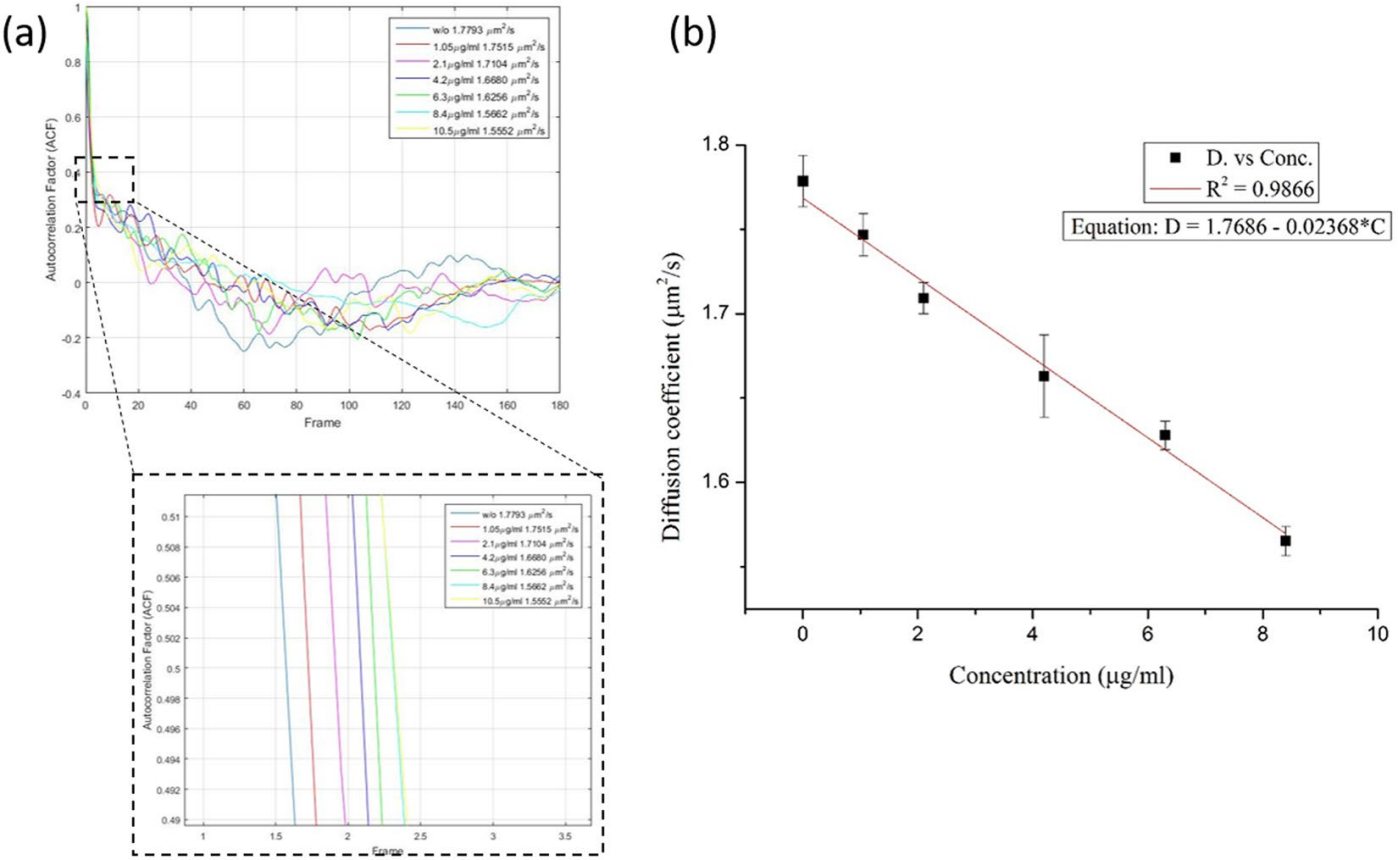
(**a**) The anti-CRP-coated nanobeads’ diffusion coefficient measured by smartphone-based diffusometry in different CRP solutions. (**b**) After repeating the analysis three times, the values of the nanobeads’ diffusion coefficients in an equilibrium state showed a linear decrease in the concentration range of 1~8 µg/ml (n = 3).

Several CRP samples with concentrations of 0, 1.05, 2.1, 4.2, 6.3, 8.4, and 10.5 µg/mL were prepared for this experiment. The CRP (C4063, Sigma) with original concentration of 53 μg/ml diluted in these basic solutions with different concentrations has been prepared for further study. The prepared anti-CRP-coated nanobeads were mixed with CRP samples. The mixtures were sandwiched by two cover glasses with a parafilm spacer. The CRP and anti-CRP-coated nanobeads were allowed to interact for 10 min, and the cover glasses sandwiching the mixture were loaded into the tray of the add-on system. Subsequently, sequential images of the fluorescent nanobeads were recorded by the smartphone for further FCS analysis. The nanobeads’ diffusion coefficients in different CRP concentration samples were measured as depicted in Fig. 5(a). When the nanobeads were suspended in various CRP solutions, the nanobeads’ diffusion coefficients showed a drastic reduction in an equilibrium state.

After the tests had been performed three times, a linearly fitted curve of the CRP concentrations versus the diffusion coefficients of the nanobeads at equilibrium was plotted as shown in Fig. 5(b). The coefficient of determination *R*^2^ of the linear-fitted line was approximately 98%, which indicates that the equilibrium diffusion coefficient of the nanobeads was linearly related to the CRP concentration in the range of 1~8 µg/ml.

## Conclusions

In summary, we developed a smartphone-based diffusometry, which was based on the analytical method of fluorescence correlation spectrometry, to measure the nanobeads’ diffusion coefficients in a solution. By this novel technique, the CRP concentration can then be obtained. The antibody-antigen interactions could be determined by directly measuring the diffusivity of the nanobeads in a 10-min period. At higher concentrations of the antigens, the antibody-modified immunobeads yielded lower diffusion coefficients in the sensing range. Furthermore, a linear sensing range of 1~8 µg/mL using the smartphone-based diffusometry was found in this study.
